# The Good Mentee

**DOI:** 10.1055/a-2505-7693

**Published:** 2025-01-21

**Authors:** Joon Pio Hong

**Affiliations:** 1 Department of Plastic and Reconstructive Surgery, Asan Medical Center, University of Ulsan College of Medicine, Seoul, Republic of Korea

**Figure:**
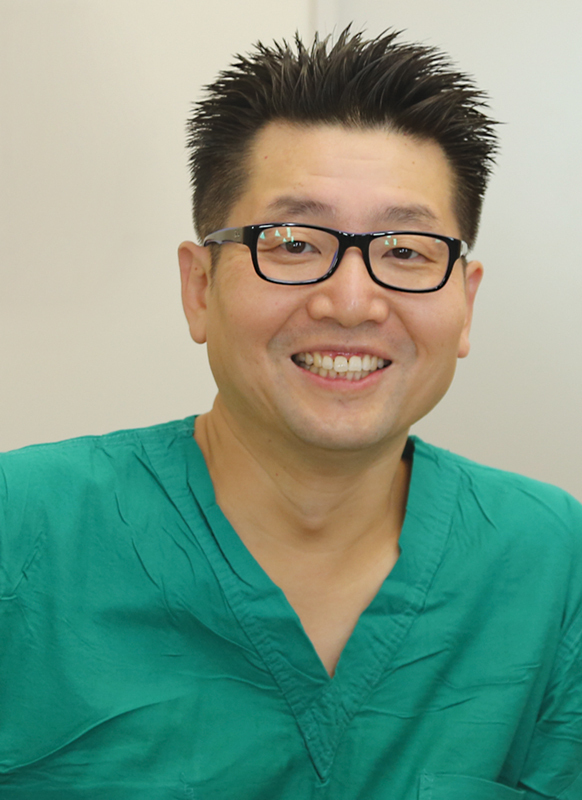
Joon Pio Hong: Editor-in-Chief

According to the Merriam-Webster dictionary, the definition of “mentee” is one who is being mentored. In the last editorial, I wrote about what it means to be a good mentor like Mentor from Odyssey who provided wisdom, teaching, and trust ultimately transcending beyond into a true friendship. 1 However, from a mentor's perspective, finding mentees who can evolve into lifelong friends is no easy task. Mentors are human beings too, with feelings and emotions. The mentor–mentee relationship, like any other, thrives on mutual respect, understanding, and effort. I sometimes encounter a mentor who talks about how great their mentees are. Then, I wonder what makes a good mentee.

In the medical field, as in any highly competitive environment, we are often unaccustomed to criticism and rarely trained to embrace new ideas. Instead, we are taught to replicate what works. This rigidity can make the field conservative and resistant to change. However, one of the key traits mentors seek in mentees is the potential for growth, which often requires the ability to adapt. Part of a mentor's role is to guide their mentee through this process of change. 2 3 4 We expect the mentees to be receptive to feedback and constructive criticism. Thus, the essential attitude expected in a mentee is being open-minded. Some mentors even encourage their mentees to challenge what they are taught—when done respectfully and with due diligence. A healthy challenge reflects an eagerness to learn, whereas baseless opposition signals ignorance and wastes valuable time. When mentees engage thoughtfully, discussions can lead to deeper learning and mutual discovery, often sparking collaborative efforts, such as research projects, to explore new ideas together.

Trust is a cornerstone of any mentor–mentee relationship. It is not unilateral but bilateral, requiring honesty and transparency from both sides. Mentees, in particular, must be forthright about their mistakes. Mistakes, and even failures, are a natural part of the learning process—especially for trainees, such as surgeons. 5 Overcoming the fear of failure and admitting errors with courage demonstrates humility and maturity. Sometimes an “I don't know,” rather than pretending to know, reflects honesty, humility, and a genuine willingness to learn. Transparent communication fosters trust, creates an environment for constructive guidance, and shows a mentee's willingness to take responsibility for their decisions and actions. This sincerity inspires mentors to invest more deeply in their mentees' growth and success.

Gratitude is another essential component of a strong mentor–mentee bond. Expressing appreciation for a mentor's time, insights, and efforts strengthens the relationship and encourages ongoing support. While it is a mentor's job to teach, genuine gratitude can transform this professional obligation into a personal commitment to the mentee's success. Mentors are more likely to provide candid feedback and go the extra mile when they know their efforts are valued. Mentoring is not just about doing the job and worrying about hurting feelings, but it is about genuine care. Any teacher would not want to get into trouble being radically candid unless the mentor knows that the mentee is ready to listen and absorb.

For those seeking a true mentor, self-reflection is key. Ask yourself if you are ready to embrace mentorship. I often tell my mentees that life offers two types of choices: those where you can influence the outcome and those where you cannot. If you feel you lack a good mentor, focus on the areas you can change—starting with yourself. Whether you meet a teacher or a lifelong friend who transcends the traditional mentor–mentee relationship, it all begins with you. Mentees can inspire teachers to become great mentors. if you do not find a true mentor during your journey, cultivating open-mindedness, proactive learning, honesty, resilience, and gratitude will still guide you toward growth and success. One day, these qualities will make you a role model for your own mentees.

Mentor–mentee relationship is a dynamic, reciprocal relationship that requires effort and intentionality from both parties. Mentees must not only seek guidance but also actively cultivate qualities that make them deserving of mentorship. Menteeship is a call to action for self-improvement, a challenge to overcome fear, and an invitation to foster meaningful human connections.
